# The Impact of Different Exercise Modes in Fitness and Cognitive Indicators: Hybrid versus Tele-Exercise in Patients with Long Post-COVID-19 Syndrome

**DOI:** 10.3390/brainsci14070693

**Published:** 2024-07-11

**Authors:** Vasileios T. Stavrou, George D. Vavougios, Kyriaki Astara, Dimitrios S. Mysiris, Glykeria Tsirimona, Eirini Papayianni, Stylianos Boutlas, Zoe Daniil, Georgios Hadjigeorgiou, Panagiotis Bargiotas, Konstantinos I. Gourgoulianis

**Affiliations:** 1 Laboratory of Cardio-Pulmonary Testing and Pulmonary Rehabilitation, Respiratory Medicine Department, Faculty of Medicine, University of Thessaly, 41100 Larissa, Greece; dantevavougios@hotmail.com (G.D.V.); zdaniil@uth.gr (Z.D.); kgourg@uth.gr (K.I.G.); 2 RespiHub, ONISLOS-MSCA COFUND, Department of Neurology, Medical School, University of Cyprus, 2029 Nicosia, Cyprus; bargiotas.panagiotis@ucy.ac.cy; 3 Department of Neurology, Medical School, University of Cyprus, 2029 Nicosia, Cyprus; hadjigeorgiou.georgios@ucy.ac.cy; 4 Department of Neurology, 417 Army Equity Fund Hospital (NIMTS), 11521 Athens, Greece; kyriakiastara@gmail.com; 5 Department of Neurology, Faculty of Medicine, University of Thessaly, 41100 Larissa, Greece; dim_mysiris@hotmail.com; 6 Respiratory Medicine Department, Faculty of Medicine, University of Thessaly, 41100 Larissa, Greece; glukatsirimona@gmail.com (G.T.); eirinipapayianni@gmail.com (E.P.); sboutlas@gmail.com (S.B.)

**Keywords:** COVID-19, hybrid exercise, tele-exercise, cognitive, tele-rehabilitation

## Abstract

The purpose of our study was to obtain evidence that an unsupervised tele-exercise program (TE_group_) via an online platform is a feasible alternative to a hybrid mode of supervised and unsupervised exercise (HE_group_) sessions for improving fitness indexes, respiratory and cognitive functions, and biomarkers of oxidative stress in patients recovering from COVID-19. Forty-nine patients with long post-COVID-19 were randomly divided into two groups (HE_group_: n = 24, age 60.0 ± 9.5 years versus TE_group_: n = 25, age 58.7 ± 9.5 years). For each patient, we collected data from body composition, oxidative stress, pulmonary function, physical fitness, and cognitive function before and after the 12-week exercise rehabilitation program (ERP). Our data showed differences in both groups before and after 12-week ERP on fitness indicators, body composition, and pulmonary function indicators. Our findings demonstrated differences between groups after 12-week ERP on adjustment in the domains of cognitive function (HE_group_ increased the “visuospatial” domain: 3.2 ± 1.1 versus 3.5 ± 0.8 score, *p* = 0.008 and TE_group_ increased the “memory” domain: 3.3 ± 1.0 versus 3.8 ± 0.5 score, *p* = 0.003; after 12-week ERP showed differences between groups in domain “attention” TE_group_: 4.8 ± 1.5 versus HE_group_: 3.6 ± 1.8 score, *p* = 0.014) and the diffusing capacity for carbon monoxide (HE_group_ increased the percent of predicted values at 0.5 ± 32.3% and TE_group_ at 26.0 ± 33.1%, *p* < 0.001). These findings may be attributed to the different ways of learning exercise programs, resulting in the recruitment of different neural circuits.

## 1. Introduction

The severe acute respiratory syndrome coronavirus 2 (SARS-CoV-2) manifests multi-systemically, adversely affecting a survivor’s health well beyond its acute phase. The residual symptoms affect principally the respiratory and central nervous systems, including breathlessness, dyspnea associated with additional oxygen requirements, mood disorders, sleep disorders, chronic fatigue, cognitive impairment, and sarcopenia [1]. A wide range of these persistent symptoms and conditions, which can last for weeks, months, or even years after Coronavirus disease 2019 (COVID-19), are defined as long-post-Coronavirus disease 2019 (long-post-COVID-19) syndrome. The most reported symptoms of long COVID-19 symptoms that have been identified are fatigue, cognitive difficulties (referred to as “brain fog”), post-exertional malaise, and dysautonomia [2]. The mechanisms of cognitive dysfunction following SARS-CoV-2 infection are not yet understood, although studies have shown prolonged neuroinflammatory responses and structural abnormalities in the brains of patients [3]. According to Aly and Rosen [3], cognitive impairment may be due to a dysfunctional hypothalamic–pituitary response and dysfunction in vagal signaling.

Physical activity impact of physical activity and exercise (supervised and/or unsupervised) in the post-Coronavirus disease 2019 (post-COVID-19) specific residual symptoms relate to reduced dyspnea during physical exercise and increase estimated oxygen uptake [4] and improve cognitive functioning [5]. Regular exercise promotes the growth and survival of neurons, fostering neuroplasticity—the brain’s ability to reorganize and form new neural connections. This phenomenon is particularly pronounced in brain regions crucial for learning and memory, such as the hippocampus [6], while physical activity influences the production and release of neurotransmitters, including dopamine, serotonin, and brain-derived neurotrophic factor, which play pivotal roles in mood regulation, stress resilience, and cognitive function [7]. Moreover, exercise exerts a positive impact on cardiovascular health, enhancing blood flow to the brain and bolstering the integrity of cerebral blood vessels. Optimal vascular function is vital for delivering oxygen and nutrients to brain cells, thereby supporting cognitive processes [8]. Exercise promotes mitochondrial biogenesis and function, fortifying cellular resilience against oxidative stress and metabolic dysfunction [9]. Therefore, rehabilitation should approach several systems to ameliorate the detrimental effects of post-COVID-19 syndrome, with exercise being the fundamental element [10]. Remote modalities that promote telemedicine and telerehabilitation are gaining popularity since they offer safe and feasible means to implement care, especially during the COVID-19 pandemic and its regional restrictive legislation [11]. In addition, technological advancement has optimized the context in which telemedicine may operate. For instance, several mobile applications have been developed to provide participants with training programs and mental health advice in the post-COVID-19 era [12,13], while the effectiveness of short-term digital individualized physiotherapy and exercise interventions has also been investigated [14].

There is insufficient data in the literature about the impact of longer duration of tele-exercise programs, especially unsupervised, along with hybrid exercise programs. Thus, the purpose of our study was to obtain evidence whether an unsupervised tele-exercise program via an online platform is a feasible alternative to a hybrid mode of supervised and unsupervised exercise sessions in improving different clinical outcomes (i.e., fitness parameters, respiratory and cognitive functions, and oxidative stress biomarkers) in patients recovering from COVID-19. We hypothesize that patients will exhibit good adherence to a tele-exercise program, and this program will yield favorable outcomes on fitness indicators and fundamental respiratory parameters, comparable to traditional face-to-face rehabilitation exercise programs.

## 2. Materials and Methods

**Figure 1:**
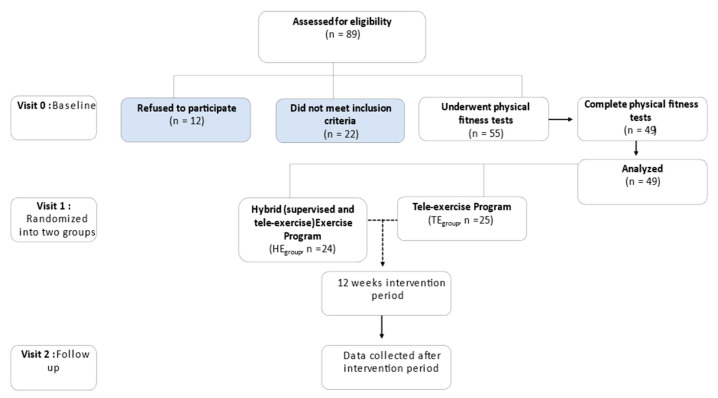
Flow study diagram. Twelve of eligibility patients refused to participate with justification “not enough time”, “discomfort feeling and exhausted”. Ten of the eligible patients did not meet the inclusion criteria, such as self-reported symptoms and incident respiratory disease, five patients had high blood pressure at rest, three patients failed the balance test, two patients lacked cooperation during spirometry, and two patients had difficulty answering the questionnaires. Seven patients were excluded after the physical fitness test: four patients shown to have decreased systolic blood pressure after a 30 s sit-to-stand test; two patients showed 6MWT, staggering, and leg cramps; and one patient gave up the trials.

Patients previously hospitalized with a SARS-CoV-2 infection (Figure 1) and long-post-COVID-19 condition [2] were recruited three months after discharge from the hospital. The patients were randomly divided into two groups (Table 1) using the block randomization technique [15]: Hybrid exercise group (HE_group_), with a twice-per-week supervised exercise in the Laboratory of Cardio-Pulmonary Testing and Pulmonary Rehabilitation Center of University of Thessaly and a once-a-week tele-exercise using the Unique Safe Tele-Exercise Project platform (USTEP, https://ustep.gr); and the tele-exercise group (TE_group_), with a three-times-per-week tele-exercise using the USTEP platform. 

Inclusion criteria included the following:

◦ Age ≥20 to ≤70 years;
◦ Discharge criteria for patients with COVID-19 (National Institute of Health and Hellenic guidance for COVID-19 pneumonia diagnosis) [16];
◦ Long-post-COVID-19 condition [2].

Exclusion criteria included the following:

◦ Any contraindication for six-minute walk test (6MWT) [17];
◦ Failed the balance test stork stand with open eyes [18];
◦ Extreme obesity (body mass index ≥ 40 kg/m^2^);
◦ Any known neurodegenerative disease from the medical history or sign through neurological examination suggesting neurodegenerative disease, and musculoskeletal disability which could impair maximum exercise capacity;
◦ Active self-reported symptoms (chest pain, fatigue and/or dyspnea);
◦ Laboratory-confirmed, incident respiratory disease [19,20,21].

The study was approved by the Institutional Review Board/Ethics Committee of the University Hospital of Larissa (approval reference number: 39252/3 November 2021). All participants had provided written informed consent, in accordance with the Helsinki declaration and personal data according to the European Parliament and of the Council of the European Union [22].

### 2.1. Measurements

#### 2.1.1. Anthropometric Characteristics and Body Composition

Anthropometric characteristics and body composition were measured using standard medical equipment. Height was measured with a Seca 700 (Hamburg, Germany) without shoes. The Seca 201 (Seca, Hamburg, Germany) was used to measure Δchest, i.e., the difference between the chest circumference at maximum inhalation and exhalation, the waist–hip ratio (i.e., the ratio of the circumference of the waist at the midpoint between the lower edge of the last palpable rib and that of the hips at the top of the iliac crest) and the neck circumference (between approximately C3 and C4 cervical vertebra and, in men with a laryngeal prominence, just below the prominence) in the standing position. Body composition indices included muscle mass, percentage of body fat, visceral fat, lean body mass and total body water (Tanita MC-980, Arlington Heights, IL, USA) [23] via bioelectrical impedance. Body mass index (BMI) calculated using the formula: $\frac{body\;mass\;(kg)}{height\;(m)\;\times \;height\;(m)}$. Body composition measurements were performed by the same operators in the morning between 10:00 and 11:30, according to the manufacturer’s recommendations.

#### 2.1.2. Oxidative Stress

Biomarkers of oxidative stress were assessed using a 10 mL peripheral venous blood sample collected 40 min prior to the exercise tests. The sample was used to measure levels of reactive oxygen metabolites (d-ROMs test) and plasma antioxidant capacity (PAT test), using the Free Radical Analytical System (FRAS5, Parma, Italy), to estimate oxidative stress (d-ROMs) and quantified water-soluble antioxidant content (PAT) [23]. The measurements of oxidative stress biomarkers were performed by the same operators in the morning between 09:00 and 09:30, according to the manufacturer’s recommendations, and all patients had fasted the night before.

#### 2.1.3. Pulmonary Function Test

Pulmonary function testing was performed in the seated position using the MasterScreen-CPX pneumotachograph (VIASYS HealthCare, Germany) with standard spirometry (i.e., a procedural breath cycle with 3 phases of maximal inspiration, a secretory exhalation, and continued full exhalation to the end of the test for at least 6 s) [24] and diffusing capacity for carbon monoxide (i.e., first they take a normal resting breath, followed by a full expiration to residual volume, then they are asked to rapidly inhale the test gas to vital capacity and hold for 10 s at full lung capacity, then exhale completely, and the exhaled gas is collected for analysis after excluding the initial amount of gas from the dead space) [24]. Pulmonary function tests were performed by the same operators in the morning, according to American Thoracic Society and European Respiratory Society guidelines [24]. 

#### 2.1.4. Cognitive Assessment

Prior to the physical fitness test, all participants completed the Montreal Cognitive Assessment (MoCA) questionnaire to assess cognitive impairment [25]. MoCA, a brief neurocognitive scale validated in the Greek population, has been widely used to detect cognitive decline in populations with subjective or objective cognitive impairment due to mild cognitive impairment, dementia, or other neurodegenerative diseases [26]. The MoCA test was administered by certified examiners. 

#### 2.1.5. Physical Fitness Test

To assess the physical fitness all patients underwent a six-minute walk test (6MWT) [27,28], a handgrip strength test using the electronic dynamometer (Camry, EH 101, South El Monte, CA, USA), and a 30-s sit-to-stand test [29], according to methods that used in our previous studies [4,19,20]. 

### 2.2. Interventions Exercise Program 

The intervention rehabilitation exercise program consisted of hybrid exercise and tele-exercise, supported by the USTEP platform (https://ustep.gr). The intervention program lasted 3 months, with each patient taking part in 3 training sessions per week with a duration of approximately 60 min per training session (Table 1). The warm-up and cool-down were 5 min each and consisted of routine exercises for mobility and respiratory exercises for 2 sets of 20–30 s and 20 s of rest. The mobility exercises included (a) a child’s pose/prayer stretch, (b) a standing quadricep stretch, and (c) a doorway stretch. The respiratory exercises included (a) an overhead chest stretch from a sitting position and (b) a yawn to a smile. The intensity of the warm-up and cool-down using the Borg scale for dyspnea and leg fatigue was measured on a 1-to-3 scale. Strength exercises were performed to improve the strength of the upper and lower limbs and the intensity was calculated according to the handgrip strength test and the 30-s sit-to-stand test [19]. During the aerobic exercise, every five minutes, patients checked their heart rate and oxygen saturation and subsequently recorded the total distance covered (outdoor walking). The data was recorded using an Activity Tracker Xiaomi Mi Band 5 (Beijing, China) and a pulse oximetry JKZ-301 (JZIKI, Guangdong, China). The evaluated parameters (HR, SpO_2_, dyspnea, and leg fatigue) were uploaded to the USTEP platform at the end of each session.

### 2.3. Statistical Analysis

For the sample size calculation of this study [30], a power of 86% and a confidence interval of 95% were adopted, with an estimated value for a type 1 error of 5%, as there was no previous study investigating the effect of hybrid mode exercise (HE_group_) versus tele-exercise (TE_group_) between COVID-19 survivors. As a result, a value of 20 patients was obtained. However, because this is a new method of exercise, we recruited more patients (Figure 1). Data are presented as mean ± standard deviation (SD) and percentage (%) where appropriate. Data normality was assessed via the Kolmogorov–Smirnov one sample test. An independent samples *t*-test was used to assess differences between groups (HE_group_ versus TE_group_). A paired *t*-test was used to assess differences before and after 12-week interventions rehabilitation exercise period. Cohen’s d was calculated from the mean difference between groups (M1 and M2), and by the pooled standard deviation (SD). Cohen’s d =$\;\frac{M2-M1}{{SD}_{pooled}}$ and SD_pooled_ =$\;\sqrt{\frac{{SD1}^{2}+{SD2}^{2}}{2}}$. For all tests, a *p*-value of <0.05 was considered statistically significant. The IBM SPSS 21 statistical package (SPSS Inc., Chicago, IL, USA) was used for all statistical analyses.

## 3. Results

### 3.1. Adherence to Program

The compliance and adherence to the program for HE_group_ and TE_group_ was 85% and 87%, respectively. For the HE_group_ there was recorded in presentation for face-to-face exercise rehabilitation session. For tele-exercise rehabilitation (HE_group_ and TE_group_), we recorded the click and length of stay on individual exercise pages for each patient, according to Google Analytics. 

### 3.2. Body Composition, Oxidative Stress, Respiratory Performance, Physical Fitness, and Hemodynamic Parameters 

Table 2 shows the analysis of results between groups before and after the 12-week exercise rehabilitation period. According to Table 2, differences in body composition were observed in both groups, and more specifically, a reduction in body and visceral fat and an increase in lean body mass and muscle mass. In terms of anthropometric parameters, neck circumference and waist-to-hip ratio decreased. In addition, increases in antioxidant capacity, physical fitness, and respiratory performance were observed in both groups. After 12 weeks of exercise rehabilitation, stabilization was observed in hemodynamic parameters.

### 3.3. Oxygen Saturation

Oxygen saturation did not show statistically significant differences between groups at the baseline period or after the 12-week exercise rehabilitation period in ΔSpO_2_ [difference between rest and at the end of test (Δ) in oxygen saturation, with a pulse oximetry (SpO_2_)] before and after a 30-s sit-to-stand test. The HE_group_ fixed the desaturation after repetitions at 34.2 ± 39.1% (t_(23)_ = 2.348, *p* = 0.028) and the TE_group_ fixed the desaturation after repetitions at 57.7 ± 34.7%, (t_(24)_ = 3.006, *p* = 0.006). 

In the baseline measurement, SpO_2_ at the end of the 6MWT appeared to significantly reduce compared to the values at the start of the test in both groups, while a higher percentage of reduction in SpO_2_ was observed in the TE_group_ (−3.5 ± 3.6 versus −2.6 ± 2.5%, *p* < 0.001). After 12 weeks without a significant drop in SpO_2_ appeared comparable to the values at the start of the test in both groups (HE_group_: −2.3 ± 0.9 versus TE_group_: −2.1 ± 1.1%, *p* = 0.365).

### 3.4. Cognitive Assessment 

**Figure 2:**
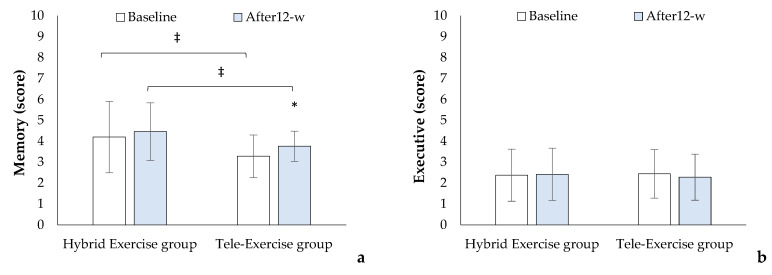

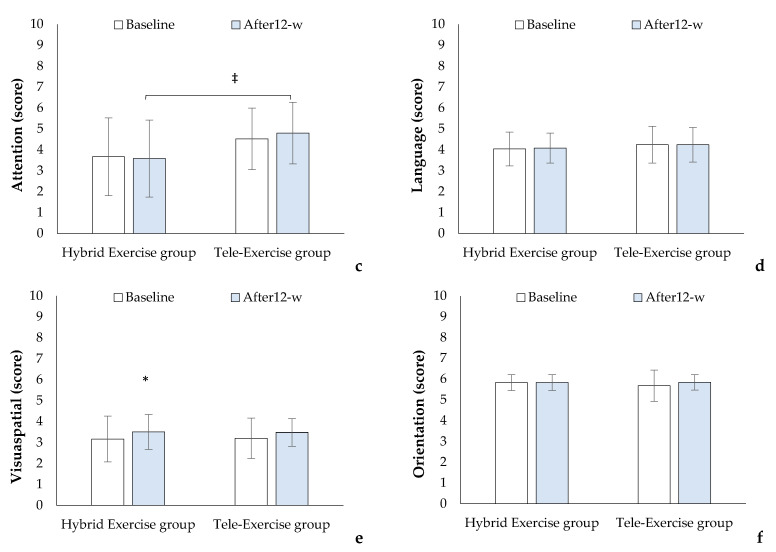
Changes in Montreal cognitive assessment (MoCA) domains: (**a**) = Memory (Cohen’s d: Baseline = 0.65; After 12 weeks = 0.63), (**b**) = Executive (Cohen’s d: Baseline = 0.00; After 12 weeks = 0.09), (**c**) = Attention (Cohen’s d: Baseline = 0.48; After 12 weeks = 0.72), (**d**) = Language (Cohen’s d: Baseline = 0.24; After 12 weeks = 0.13), (**e**) = Visuaspatial Cohen’s (d: Baseline = 0.00; After 12-week = 0.00), and (**f**) = Orientation Cohen’s (d: Baseline = 0.18; After 12 weeks = 0.00). Cohen’s d effect size classification: d ≥ 0.00 to < 0.20 is ignored, d ≥ 0.20 to <0.50 is small, d ≥ 0.50 to <0.80 is medium, d ≥ 0.80 to <1.30 is large, and d ≥ 1.30 is very large. * *p* < 0.05 between baseline and after the 12-week rehabilitation period, ^‡^ *p* < 0.05 between groups.

At the baseline measurement, and after a 12-week exercise rehabilitation period, both groups showed differences in MoCA score (HE_group_: 23.4 ± 3.7 versus 24.4 ± 3.1 score, t_(24)_ = −2.614, *p* = 0.015, TE_group_: 23.4 ± 4.0 versus 23.9 ± 3.7, t_(23)_ = −2.505, *p* = 0.020). The Cohen’s d effect size was d = 0.00 at baseline and d = 0.15 at 12 weeks. The HE_group_ increased in the domain “visuospatial” (HE_group_: 3.2 ± 1.1 versus 3.5 ± 0.8 score t_(23)_ = −2.892, *p* = 0.008, Figure 2e) and the TE_group_ increased in the domain “memory” (3.3 ± 1.0 versus 3.8 ± 0.5 score, t_(24)_ = −3.361, *p* = 0.003, Figure 2a). After 12 weeks, differences were present between groups in the “attention” domain. The TE_group_ showed a higher score (4.8 ± 1.5 versus 3.6 ± 1.8 score t_(23)_ = −2.562, *p* = 0.014, Figure 2c) compared to the HE_group_. 

All patients at the baseline showed a statistically significant correlation detected between the following: (a) ΔSpO_2_ in 6MWT and MoCA score (r = −0.415, *p* = 0.003), domain “language” (r = −0.339, *p* = 0.019), and diffusing capacity for carbon monoxide (r = −0.491, *p* = 0.001); (b) covered meters during 6MWT and MoCA score (r = 0.385, *p* = 0.007), and domains “executive” (r = 0.385, *p* = 0.007) and “attention” (r = 0.308, *p* = 0.033). After the 12-week exercise rehabilitation period, all participants, independent of rehabilitation group, showed a statistically significant correlation between covered meters during 6MWT and MoCA score (r = 0.377, *p* = 0.008) and the domains “executive” (r = 0.313, *p* = 0.029) and “language” (r = 0.334, *p* = 0.019).

## 4. Discussion

In our study, we aimed to investigate potential differences in fitness indicators and cognitive function in previously hospitalized patients with SARS-CoV-2 infection after a 12-week exercise rehabilitation period compared to two modes of exercise: hybrid exercise versus tele-exercise. Our findings demonstrated differences in both groups before and after the 12-week exercise rehabilitation period on fitness indicators, body composition, and pulmonary function indicators. In addition, our findings demonstrated no differences between groups after 12 weeks on fitness indicators but different adjustments in the domains of cognitive function and the diffusing capacity for carbon monoxide.

Stavrou et al. [4,19] have shown that rehabilitation exercise programs improve fitness indicators in hospitalized patients with SARS-CoV-2 infection. Our study results showed that patients after the 12-week intervention period improved their physical fitness indicators. Both groups after 12-week exercise improved handgrip strength by 9%, lower limb strength (via a 30-s sit-to-stand test) by 22%, and increased the covered distance during 6MWT (HE_group_: 80.9 ± 7.3 m vs. TE_group_: 54.8 ± 7.1 m), findings consistent with previous studies [31]. In addition, patients after the rehabilitation exercise program period reduced breathlessness at the end of 6MWT by 3.3% and their desaturation at the end of 6MWT by 2.2%. These findings are likely to correlate with improvements in physical fitness, both in terms of body composition (i.e., ↓ body and visceral fat, ↑ lean body mass and muscle mass) parameters and anthropometric parameters (i.e., ↓ neck circumference and waist–hip ratio) related to the general health of the patients managing their symptoms, findings consistent with previous studies [32]. In our study, the exercise load of the rehabilitation program (aerobic and resistance training) was submaximal in both groups. According to Barbara et al. [33], previous studies by our research group [4,19] have shown that moderate-intensity aerobic endurance training is more efficient and may improve physical performance deficits in patients with post-COVID-19.

The reduction in dyspnea after PR is related to cardiorespiratory adaptations and vagus nerve function. Patients with long COVID-19 exhibit dysautonomia [34] and vagus and phrenic nerve dysfunction [35]. Previous research reported that signals transmitted through the vagus nerve stimulate central stem chemoreceptors, while the intervention program of aerobic exercise probably caused activation of anaerobic metabolism and activation of the lactic acid neutralization mechanism resulting in sodium lactate (NaC_3_H_5_O_3_) production and carbon dioxide release according to the Haldane effect [36], while the resistance exercise during the PR program probably caused an increase in the arteriovenous oxygen difference in the muscles and changes in the oxyhemoglobin saturation curve. In addition, the diffusing capacity for carbon monoxide improved in both groups after the rehabilitation period, but higher changes showed the TE_group_ by 26%. Exercise was shown to correct desaturation and improve cerebral oxygenation. These changes probably relate to increased pulmonary capillary blood volume and diffusion membrane capacity to meet exercise oxygen delivery requirements [37] and/or residual attenuation after recovery [38]. According to Vavougios et al. [39], the diffusion capacity abnormalities for carbon monoxide in surviors of COVID-19 may be implicated in the development of cognitive impairment, while desaturation and low physical condition during 6MWT may indicate overlap with post-COVID-19 fatigue [40]. 

Previous studies have shown that cognitive complaints may be far more insidious and potentially long-lasting, constituting a robust clinical manifestation in the realm of post-COVID-19 syndrome [41]. In our study, the cognitive performance of the groups significantly differed before and after PR, and we observed different changes in cognitive domains depending on the intervention group. The neurotrophic effects of exercise are well documented, as there have been reported phenomena of neuroplasticity and structural changes, such as increased gray matter volume in the frontal and hippocampal regions and reduced damage to gray matter, while exercise facilitates the release of neurotrophic factors [9]. Our results reveal improved cognitive function, findings consistent with previous studies [42]. To our knowledge, as far as the significant differences in particular domains are concerned, our study was the first to indicate some between the two groups, despite not being powered to detect them. Although it could be attributed to the different ways of teaching and demonstrating the exercise programs, resulting in the recruitment of different neural circuits, more extended studies incorporating detailed cognitive assessment may elucidate the interrelationship of exercise and cognition. It could be hypothesized that the overall improvement in cognitive status is probably associated with cerebral reserves and neural circuits, while memory improvement via tele-exercise is related to a neural compensation mechanism that permits the performance of complex activities [43]. Moreover, exercise shields blood–brain barrier integrity, which is strongly associated with cognitive status [44], by enhancing tight junction protein expression and reducing inflammatory processes [45]. In addition, exercise appears to have neuroprotective properties by increasing both cerebral blood flow and the content of neurotransmitters associated with cortical arousal at the tissue level. Exercise may contribute unique benefits to the brain and counteract many aspects of decline in the brain’s environment, while long-term exercise provides the greatest and longest-lasting benefits [46], while the intensity of exercise shows a significant relationship with cognitive functions [47]. In addition, exercise promotes mitochondrial biogenesis and function, fortifying cellular resilience against oxidative stress and metabolic dysfunction [9], findings consistent with our study, which showed an improvement in antioxidant capacity in both groups after the rehabilitation period.

### 4.1. Strength and Limitations of Study

Our study should be interpreted within the context of its strengths and limitations. The sample size was negatively influenced by COVID-19 restriction legislation imposed in Greece. Other sample characteristics, like average age and BMI, may pose intrinsic confounding factors, as both groups contained merely middle-aged participants, most of whom were obese. However, to our knowledge, this is the first study that examined the chronic effects of exercise in such an extended follow-up period. Another important limitation is that specific results may not extrapolate to the whole context of adaptation. For instance, as far as hemodynamic parameters are concerned, only SBP was found to be significantly different, indicating inadequate autonomic adaptation. Conversely, fitness indicators were more consistently associated with adaptive changes due to exercise modes in both groups. In addition, there were no untoward incidents or concerns about the safety of participants in either type of exercise, while high adherence to the tele-exercise program was recorded. Lastly, it should be noted that our study was not able to uncover deficits in specific cognitive domains and causally correlate them to exercise benefits. More extended studies, with the ability to detect interarm differences in cognitive status, may elucidate the neurotrophic effects of exercise.

### 4.2. Recommendations for Future Research

Our study showed interesting results before and after 12 weeks of exercise rehabilitation on fitness indicators, body composition and lung function indicators, and cognitive function in both groups, despite the limitations mentioned above. However, our recommendations for future research are to research the interaction between cognitive function and cerebral oxygenation before and after acute or long-term exercise periods.

## 5. Conclusions

To conclude, the 12-week exercise program enhanced fitness indicators and cognition in both groups. However, they differed in the effects of cognitive domains, with the hybrid group being favored in the visuospatial domain and the tele-exercise one in memory. These findings may be due to different ways of learning exercise programs, probably by recruiting different neural circuits. Participants in the tele-exercise program showed high adherence with no adverse events and could be an effective and beneficial low-cost way to promote exercise in patients who have difficulty or no access to rehabilitation centers, eliminating geographical barriers and allowing individuals to access exercise sessions from the comfort of their homes. Larger-scale studies powered to detect interarm differences in cognitive status may provide further details regarding the causal interrelationship of exercise and cognition. 

## Figures and Tables

**Table 1:** Rehabilitation exercise program.

Weeks	Aerobic Exercise ^a^			Strength Exercise				
	Intensity ^b^	Duration ^c^	Sets	Resistance ^d^	Sets	Repetitions	Rest ^e^	Exercise Type ^f^
1st	90%	30 min	1	Body mass	2	6	40 s	F1, F2, F5, F6
2nd	90%	30 min	1	Body mass	2	6	40 s	F1, F2, F5, F6
3rd	95%	30 min	1	Body mass	2	8	40 s	F1, F2, F5, F6
4th	95%	30 min	1	Body mass	2	8	50 s	F1, F2, F3, F5, F6
5th	100%	30 min	1	Body mass	2	10	50 s	F1, F2, F3, F5, F6
6th	100%	30 min	1	Body mass	2	10	50 s	F1, F2, F3, F5, F6
7th	105%	30 min	1	Body mass	3	8	40 s	F1, F2, F3, F4, F5, F6
8th	105%	30 min	1	Body mass	3	8	40 s	F1, F2, F3, F4, F5, F6
9th	110%	30 min	1	Body mass	3	10	50 s	F1, F2, F3, F4, F5, F6, F8
10th	110%	30 min	1	Body mass	3	10	50 s	F1, F2, F3, F4, F5, F6, F8
11th	110%	30 min	1	Body mass	3	12	40 s	F1, F2, F3, F4, F5, F6, F7, F8
12th	110%	30 min	1	Body mass	3	12	40 s	F1, F2, F3, F4, F5, F6, F7, F8

Abbreviations: min = minute; s = seconds; ^a^ Aerobic exercise: HE_group_: interval cycle ergometer twice a week (face-to-face exercise) and outdoor walking once a week (tele-exercise); TE_group_: outdoor walking three times a week (tele-exercise); ^b^ The intensity of aerobic exercise was calculated according to HR_peak_ during 6MWT; ^c^ The total duration of each session was 30 min for both groups. The aerobic exercise (cycle ergometer) with face-to-face method, was performed with 30 s of exercise and 30 s of rest (interval), with self-reported feelings of dyspnea and leg fatigue according to CR-10 Borg scales on 6 score, respectively. The aerobic exercise with the tele-exercise method, was performed while continuously walking on a flat, hard surface, without differences in altitude (uphill or downhill). The exercise took place away from main roads to avoid exhaust fumes and at a time when the humidity was not high. The self-reported feelings of dyspnea and leg fatigue according to CR-10 Borg scales on 5 score, respectively; ^d^ Strength exercises were performed using functional circuit training and the resistance for each participant was their body weight. Every two weeks the body mass was measured, and we adjusted the sets and repetitions; ^e^ Resting between sets; ^f^ The strength exercises used were as follows: F1 = seated leg raises; F2 = abdominal crunch; F3 = squats; F4 = push-ups; F5 = lunges; F6 = chair dip; F7 = jumping jack; F8 = mountain climbers.

**Table 2:** Results of analysis between groups, before and after the intervention period. Data are expressed as numbers, means ± standard deviations and percentages. The statistically significant differences before and after the intervention period and between groups are marked with symbols (between baseline and after the 12-week rehabilitation period: ^†^ *p* < 0.001 and * *p* < 0.05; between groups: ^#^ *p* < 0.05).

		Hybrid Exercise Group			Tele-Exercise Group			Cohen’s d	
		Baseline	After 12 w	% Changes within Group	Baseline	After 12 w	% Changes within Group	Baseiline	After 12 w
Age	years	60.0 ± 9.5	-	-	58.7 ± 9.5	-	-	0.13	-
Sex (Female)	n	6	-	-	8	-	-	-	-
Body mass index	kg/m^2^	30.5 ± 4.0	30.7 ± 4.0	3.4 ± 4.3	27.9 ± 4.9	28.9 ± 4.8 ^#^	4.9 ± 3.9	0.58	0.53
Lean body mass	kg	62.1 ± 6.5	62.4 ± 6.5	0.4 ± 3.2	58.7 ± 7.1	59.9 ± 7.4	1.8 ± 3.1	0.50	0.36
Body fat	%	32.9 ± 9.2	30.8 ± 8.5 ^#^	−7.7 ± 12.7	32.7 ± 9.6	31.0 ± 9.0	−1.0 ± 20.1	0.02	0.03
Muscle mass	kg	29.8 ± 4.5	33.5 ± 7.4 ^#^	8.0 ± 17.6	29.7 ± 4.3	32.8 ± 13.3	7.0 ± 38.9	0.02	0.06
Visceral fat	score	14.1 ± 4.3	13.6 ± 4.3	−4.0 ± 11.7	12.3 ± 4.2 *	11.2 ± 4.1 ^#^	−7.0 ± 22.2	0.42	0.57
Neck circumference	cm	40.0 ± 3.5	38.1 ± 4.2 ^#^	−6.5 ± 7.0	38.2 ± 3.8	37.9 ± 3.6	−4.3 ± 3.5	0.49	0.28
Waist–hip ratio	m	1.0 ± 0.1	0.9 ± 0.1	−5.3 ± 6.1	1.0 ± 0.1	0.9 ± 0.2	−5.1 ± 3.8	0.00	0.00
Δchest	cm	4.0 ± 2.2	5.8 ± 2.0 ^#^	33.1 ± 20.9	5.2 ± 1.6	5.6 ± 1.4	10.3 ± 57.8	0.62	0.12
d-ROMs	U. carr.	337.7 ± 76.3	376.5 ± 102.4	8.7 ± 35.6	302.8 ± 77.0	316.3 ± 70.2	20.6 ± 39.8	0.46	0.69
PAT	U. cor.	2671.9 ± 400.1	2823.0 ± 537.1	7.6 ± 27.7	2586.7 ± 414.1	2786.1 ± 365.4	12.5 ± 44.6	0.21	0.08
FEV_1_	% of predicted	90.7 ± 18.5	91.7 ± 19.9	6.3 ± 25.8	92.4 ± 16.5	100.2 ± 15.2 ^#^	−6.3 ± 25.8	0.10	0.48
FVC	% of predicted	90.5 ± 16.6	91.9 ± 18.5	6.6 ± 26.6	93.0 ± 15.3	99.2 ± 14.3 ^#^	6.6 ± 26.6	0.16	0.44
PEF	% of predicted	106.4 ± 23.8	102.9 ± 26.0	−0.6 ± 31.5	106.8 ± 25.9	113.2 ± 25.3	0.6 ± 31.5	0.02	0.40
DLCO	% of predicted	77.3 ± 13.5	80.9 ± 17.7	0.5 ± 32.3	67.7 ± 16.6 ^#^	79.7 ± 15.2 ^†^	26.0 ± 33.1	0.63	0.07
Handgrip	kg	31.7 ± 10.1	33.4 ± 10.0 *	9.0 ± 7.2	32.5 ± 9.0	33.5 ± 10.1 *	9.1 ± 7.5	0.08	0.01
30 s sit-to-stand	repetitions	11.4 ± 3.4	13.9 ± 2.5 ^†^	19.8 ± 15.9	12.4 ± 3.0	14.7 ± 4.3 *	23.9 ± 7.7	0.31	0.22
6MWT	m	440.5 ± 95.3	521.4 ± 88.0 ^†^	15.5 ± 12.4	500.8 ± 114.9	555.6 ± 107.9 ^†^	13.7 ± 12.8	0.57	0.35
6MWT	% of predicted	84.4 ± 16.8	99.8 ± 13.1 ^†^	-	87.1 ± 25.9	101.9 ± 16.5 ^†^	-	0.12	0.14
Leg fatigue _BS-resting_	score	0.5 ± 0.8	0.1 ± 0.3 *	−3.2 ± 1.9	0.7 ± 0.9	0.7 ± 1.3	-	0.24	0.64
Leg fatigue _BS-end 6MWT_	score	1.3 ± 1.2	1.0 ± 1.0	1.7 ± 0.9	1.8 ± 1.4	1.4 ± 2.2	0.6 ± 0.1	0.38	0.23
Dyspnea _BS-resting_	score	0.3 ± 0.6	0.1 ± 0.3 *	0.2 ± 0.4	0.5 ± 0.8	0.6 ± 1.0	0.4 ± 0.6	0.28	0.68
Dyspnea _BS-end 6MWT_	score	1.4 ± 1.6	0.5 ± 0.8 *	−3.9 ± 1.1	2.0 ± 1.7	1.0 ± 1.4 *	−3.5 ± 0.9	0.36	0.44
SBP _resting_	mmHg	136.2 ± 15.9	128.0 ± 9.1 *	−5.5 ± 12.6	125.4 ± 15.3	128.2 ± 13.6	−1.5 ± 14.3	0.69	0.02
SBP _end-6MWT_	mmHg	155.8 ± 15.4	151.6 ± 12.3	−2.2 ± 11.4	151.7 ± 26.8 ^#^	146.3 ± 19.8 ^#^	−6.4 ± 23.7	0.19	0.32
DBP _resting_	mmHg	83.1 ± 11.4	83.8 ± 5.6	-	78.7 ± 7.9	81.0 ± 7.6	−2.5 ± 9.8	0.45	0.42
DBP _end-6MWT_	mmHg	86.8 ± 9.1	87.1 ± 6.9	0.7 ± 12.7	85.2 ± 10.3	81.3 ± 9.2	1.9 ± 26.1	0.16	0.71
HR _resting_	bpm	77.9 ± 16.0	76.5 ± 11.2	−2.3 ± 21.8	79.2 ± 13.6	76.8 ± 12.9	−4.6 ± 15.1	0.09	0.47
HR _end-6MWT_	bpm	113.2 ± 19.0	110.6 ± 16.8	−6.5 ± 12.4	123.8 ± 18.4	113.5 ± 17.2	−6.0 ± 28.7	0.57	0.17

Abbreviations: 6MWT = six-minute walking test; BS = Borg Scale; DBP = diastolic blood pressure; DLCO = diffusing capacity for carbon monoxide; d-ROMs = reactive oxygen metabolites’ levels; FEV_1_ = forced expiratory volume in 1st sec; FVC= forced vital capacity; HR = heart rate; PAT = plasma antioxidant capacity; PEF = peak expiratory flow; SBP = systolic blood pressure; Δchest = chest circumference difference between maximal inhalation and exhalation. Cohen’s d effect size classification: d ≥ 0.00 to < 0.20 is ignored, d ≥ 0.20 to <0.50 is small, d ≥ 0.50 to < 0.80 is medium, d ≥ 0.80 to < 1.30 is large, and d ≥ 1.30 is very large.

## Data Availability

The data presented in this study are available upon request from the corresponding author. Due to the applicable data protection legislation in Greece (Law 4624/2019), the data are not publicly available.
